# Development and Application of InDel Markers for *Capsicum* spp. Based on Whole-Genome Re-Sequencing

**DOI:** 10.1038/s41598-019-40244-y

**Published:** 2019-03-06

**Authors:** Guangjun Guo, Genlian Zhang, Baogui Pan, Weiping Diao, Jinbing Liu, Wei Ge, Changzhou Gao, Yong Zhang, Cheng Jiang, Shubin Wang

**Affiliations:** 1 Institute of Vegetable Crops, Jiangsu Academy of Agricultural Sciences/Jiangsu Key Laboratory for Horticultural Crop Genetic Improvement, Nanjing, Jiangsu 210014 China; 2 College of Horticulture, Nanjing Agricultural University, Nanjing, 210095 China; 3 College of Horticulture, Henan Agricultural University, Zhengzhou, 450002 China

## Abstract

Genome-wide identification of Insertion/Deletion polymorphisms (InDels) in *Capsicum* spp. was performed through comparing whole-genome re-sequencing data from two *Capsicum* accessions, *C. annuum* cv. G29 and *C. frutescen*s cv. PBC688, with the reference genome sequence of *C. annuum* cv. CM334. In total, we identified 1,664,770 InDels between CM334 and PBC688, 533,523 between CM334 and G29, and 1,651,856 between PBC688 and G29. From these InDels, 1605 markers of 3–49 bp in length difference between PBC688 and G29 were selected for experimental validation: 1262 (78.6%) showed polymorphisms, 90 (5.6%) failed to amplify, and 298 (18.6%) were monomorphic. For further validation of these InDels, 288 markers were screened across five accessions representing five domesticated species. Of these assayed markers, 194 (67.4%) were polymorphic, 87 (30.2%) monomorphic and 7 (2.4%) failed. We developed three interspecific InDels, which associated with three genes and showed specific amplification in five domesticated species and clearly differentiated the interspecific hybrids. Thus, our novel PCR-based InDel markers provide high application value in germplasm classification, genetic research and marker-assisted breeding in *Capsicum* species.

## Introduction

Desirable as both vegetable and spice, pepper (*Capsicum* spp. L.), native to South and Central America, is an economically important genus in Solanaceae family^1,2^. Thirty-one species in the genus *Capsicum* have been identified^3^. Among these, five have been domesticated including *C. annuum*, *C. chinense* Jacq., *C. baccatum*, *C. pubescens* Ruiz & Pavon and *C. frutescen*s^4,5^. *C. annuum* is the predominant species planted around the world, and together with closely related *C. chinense* and *C. frutescens*, is part of what has been described known as the *C. annuum* complex^6^. A comparison of morphological traits has been the traditional approach for determining genotypes and assessing genetic diversity^7^. Nevertheless, phenotypic evaluation is easily affected by environmental factors and is not an accurate method for identification of closely related genotypes^8,9^. More recently, application of DNA markers has allowed for better discrimination among the species in existing complexes^10–12^. In multiple crops, DNA markers have played a vital role in DNA fingerprinting, genetic diversity analysis, as well as variety identification and marker-assisted breeding^13–16^.

During the last several decades, the molecular DNA markers of *Capsicum* have experienced three stages of development as in other organisms^9^. As the first and second-generation DNA markers, restriction fragment length polymorphism (RFLP), amplified fragment length polymorphism (AFLP), random amplified polymorphic DNA (RAPD), simple sequence repeats (SSR) and their derived methods have been extensively applied to a variety of genetic studies in pepper^17–24^. More recently, single nucleotide polymorphisms (SNPs) and insertion/deletion polymorphism (InDels), have become more commonly applied as the third-generation markers in pepper^9,25–27^.

Compared with the requirement of special equipment system for SNP detection^28^, codominant InDels technology is user-friendly and indeed advantageous in some genetic analyses, especially in marker-assisted selection (MAS) breeding^9,29,30^. With the development and decreasing cost of the second and third generation sequencing technology, InDels have been identified and developed extensively through re-sequencing and have become a valuable resource for the study of various organism, especially plants and animals^30–33^. The publication of pepper genomic date has provided an important platform for the detection and development of genome-wide InDels^2,34^. In *Capsicum*, multiple genetic maps were constructed with InDels based on intraspecific or interspecific populations^9,27,33^. In addition, InDels markers were used for QTL analysis in pepper, such as CMV resistance and initiation of flower primordia^25,28^. However, discovery efforts for InDels have lagged significantly behind those for SNPs, and relatively few InDels have been developed and applied in pepper^28,35,36^, nor have they been used with any frequency for pepper variety characterization or germplasm diversity assessment.

The purpose of the present study was to discover and develop stable and practical InDels based on re-sequencing data from *C. annuum* cv. G29 and *C. frutescens* cv. PBC688, as compared to a reference genome sequence, which could be detected with simple procedures based on size separation. Furthermore, identified polymorphic InDels among five domesticated species including two re-sequencing accessions and five additional ones. These reliable polymorphic InDels will become a useful resource for the *Capsicum* species identification, genetic relationship analysis and hybridization studies.

## Materials and Methods

### Plant materials

Two pepper lines *C. annuum* cv. G29 and *C. frutescens* cv. PBC688 were selected for re-sequencing in this study. The former is a sweet line ssceptible to CMV, but with excellent horticultural traits, while the latter represents a wild small-fruited hot accession highly resistant to CMV. Among the 176 accessions introduced by Dr. W.P Diao from the National Plant Germplasm System (NPGS) of United States Department of Agriculture (USDA) in 2015, we selected 63 accessions representing five domesticated species of *Capsicum* (Table 1). Five accessions each representing one domesticated species: PI 224408 (2), PI 439512 (15), PI 441620 (24), PI 441539 (46), and PI 585277 (59) were carefully chosen for InDel polymorphism validation of inter-species together with G29 and PBC688. Two *C. annuum* accessions, G29 and G43, together with two *C. frutescens* PBC688 and PI 439512 (15) were tested for InDel intra-species polymorphism. All 63 accessions were used for validation of inter-species InDel polymorphism.

**Table 1:** The 63 accessions representing 5 domesticated species of *Capsicum*.

Serial	Accession ID	Accession name	Origin	Source	Species
1	PI 194881	EBONY	United States, New York	NPGS	*C. annuum*
2	PI 224408	No.1546	Mexico	NPGS	*C. annuum*
3	Grif 9108	BG-639	Mexico	NPGS	*C. annuum*
4	PI 368479	GREKA PIPERKA II	Former Serbia and Montenegro	NPGS	*C. annuum*
5	PI 260449	COL NO 187	Argentina	NPGS	*C. annuum*
6	PI 338490		Bulgaria	NPGS	*C. annuum*
7	PI 592831	SWEET CHOCOLATE	United States, Minnesota	NPGS	*C. annuum*
8	PI 203524	No.3	Cuba	NPGS	*C. annuum*
9	PI 201239	CHILE ARCHO SAN LUIS	Mexico	NPGS	*C. annuum*
10	PI 634826	GREENLEAF TABASCO	United States, Alabama	NPGS	*C. frutescens*
11	PI 441649	BGH 1797	Brazil, Minas Gerais	NPGS	*C. frutescens*
12	PI 631144	chile nan	Guatemala, Jutiapa	NPGS	*C. frutescens*
13	PI 593924	WWT-1336	Ecuador	NPGS	*C. frutescens*
14	PI 487623		Costa Rica	NPGS	*C. frutescens*
15	PI 439512	Rat chili	Mexico	NPGS	*C. frutescens*
16	PI 439521	834	Solomon Islands	NPGS	*C. frutescens*
17	PI 585251	Ecu 2239	Ecuador, Manabi	NPGS	*C. frutescens*
18	PI 194260	1SCA	Ethiopia	NPGS	*C. frutescens*
19	Grif 9319	14031	Costa Rica	NPGS	*C. frutescens*
20	PI 631142	diente de perro	Guatemala, Escuintla	NPGS	*C. frutescens*
21	PI 645561	Chiang Mai #1	Thailand	NPGS	*C. frutescens*
22	PI 441652	BGH 4179	Brazil, Minas Gerais	NPGS	*C. frutescens*
23	PI 159248	1SCA	United States, Georgia	NPGS	*C. chinense*
24	PI 441620	BGH 1719	Brazil	NPGS	*C. chinense*
25	PI 224412	No.1555	Bolivia	NPGS	*C. chinense*
26	PI 152222	1SCA	Peru	NPGS	*C. chinense*
27	PI 257176	1SCA	Peru	NPGS	*C. chinense*
28	PI 543208	Aji	Bolivia	NPGS	*C. chinense*
29	PI 224449	No.1633	Peru	NPGS	*C. chinense*
30	PI 241668	1SCA	Ecuador	NPGS	*C. chinense*
31	PI 562384	RED SAVINA HABANERO	United States	NPGS	*C. chinense*
32	PI 438643	Habanero No. 44	Mexico, Yucatan	NPGS	*C. chinense*
33	PI 640902	Yellow Squash	United States	NPGS	*C. chinense*
34	PI 438636	Habanero No. 1	Mexico, Yucatan	NPGS	*C. chinense*
35	PI 653672	Peru-7209	Costa Rica	NPGS	*C. chinense*
36	Grif 9238	13978	Costa Rica	NPGS	*C. chinense*
37	Grif 9182	Grif 9182	Colombia	NPGS	*C. chinense*
38	PI 159236	30040	United States, Georgia	NPGS	*C. chinense*
39	PI 656271	6123	Costa Rica	NPGS	*C. chinense*
40	Grif 9261	Honduras-11058	Costa Rica	NPGS	*C. chinense*
41	PI 241650	No.1236	Peru	NPGS	*C. chinense*
42	PI 593612	30062	United States, New Mexico	NPGS	*C. chinense*
43	PI 159234	No.4658	United States, Georgia	NPGS	*C. chinense*
44	PI 653673	Grif 9302	Colombia	NPGS	*C. chinense*
45	PI 639649	WWCQ-207	Paraguay, Canendiyu	NPGS	*C. baccatum* var. *baccatum*
46	PI 441539	BGH 1036	Brazil, Minas Gerais	NPGS	*C. baccatum* var. *pendulum*
47	PI 653670	Peru-5391	Costa Rica	NPGS	*C. baccatum* var. *pendulum*
48	PI 441553	BGH 1668	Brazil, Minas Gerais	NPGS	*C. baccatum* var. *pendulum*
49	Grif 9198	Peru-5383	Costa Rica	NPGS	*C. baccatum* var. *pendulum*
50	PI 441545	BGH 1607	Brazil, Minas Gerais	NPGS	*C. baccatum* var. *pendulum*
51	PI 497972	Dedo de Moca	Brazil	NPGS	*C. baccatum* var. *pendulum*
52	PI 596058	3015	Bolivia, Chuquisaca	NPGS	*C. baccatum* var. *pendulum*
53	PI 439388	1986	Peru	NPGS	*C. baccatum*
54	PI 596055	3009	Bolivia, Chuquisaca	NPGS	*C. baccatum* var. *pendulum*
55	PI 632922	WWMC 122	Paraguay, Caazapa	NPGS	*C. baccatum* var. *baccatum*
56	PI 281300	Cristal	Argentina	NPGS	*C. baccatum* var. *pendulum*
57	PI 281320	Aji cristal	Chile	NPGS	*C. baccatum* var. *pendulum*
58	PI 441570	BGH 1785	Brazil, Minas Gerais	NPGS	*C. baccatum* var. *pendulum*
59	PI 585277	Ecu 2243	Ecuador, Carchi	NPGS	*C. pubescens*
60	Grif 1613	Grif 1613	-	NPGS	*C. pubescens*
61	PI 593623	80040	Guatemala	NPGS	*C. pubescens*
62	PI 585274	Ecu 6222	Ecuador, Napo	NPGS	*C. pubescens*
63	PI 593632	80049	Guatemala	NPGS	*C. pubescens*

### Library construction and sequencing

The CTAB extraction method was used to isolate genomic DNA from fresh leaves. High quality genomic DNA was confirmed through 1.0% agarose gel electrophoresis for library construction^37^. We constructed two paired-end libraries with 10-fold depth for each pepper line. Briefly, genomic DNA was sheared using ultrasonic to yield an average size of 500 bp DNA fragments. Then Illumina paired-end adaptors were ligated to the fragmented DNA. The ligated DNA products were selected based on the fragment size on a 2% agarose gel. Amplification of the products was performed by PCR using specific primers to form the libraries. After inspection, the resulting libraries were sequenced on an Illumina Hiseq^TM^ 2500 sequencer (Illumina Inc., San Diego, CA, USA) in the company of Biomarker Technologies. Raw reads of 2 × 100 bp were generated for the downstream analyses.

### Data filtering, alignment, variants calling

The genome sequence of *C. annuum* cv. CM334 (2.96 Mb) was obtained from the Pepper Genome Platform (PGP) (http://peppergenome.snu.ac.kr/download.php) to use as the reference. Low quality reads were filtered out using a custom C program based on the default parameters. The cleaned data were aligned to the reference pepper genome using the Burrows-Wheeler Aligner (BWA0.7.10-r789) program^38^ with the default values. The alignment results in SAM format were transformed to Binary Alignment Map (BAM) format files through SAMTools^39^. Mark Duplicates in Picard tool (v1.102) (http://broadinstitute.github.io/picard/) was applied to remove replicate reads, and the two BAM files were used for the next analyses. To reduce the inaccurate alignments, GATK Tool Kits version 3.1 was used to conduct the local realignment around the insertions and deletions, reads base quality recalibration and variant calling^40^.

### InDels flanking sequences extraction and primer design

For the identification of InDel polymorphisms between the re-sequenced PBC688 and G29, we explored the reference genome of CM334 as a ‘bridge’ to detect sequence polymorphisms between them. The single-end reads of G29 were aligned to the reference sequence of CM334 via SOAP with no gaps allowed. The aligned reads dataset was compared against the InDel polymorphism dataset identified between PBC688 and CM334. Only those InDels with identical sequences between G29 and CM334 were considered as real InDels between G29 and PBC688. Once the location of InDel polymorphisms between one re-sequenced accession and the reference was established, those between the two re-sequenced accessions are readily distinguished at corresponding positions where the second accession is identical to the reference^31^. In order to develop the InDels markers, we extracted 150-bp flanking nucleotides on two sides of an InDel to query the reference genome sequence using a simple Visual C++ script for primers design. Primer 5 (http://www.PromerBiosoft.com) was used to design PCR primers with length of 19–22 bp, Tm of 52–60 °C, and PCR products of 80–250 bp.

### Chromosomal location and genomic synteny in pepper

The chromosomal localization of InDel markers was acquired from the CM334 genome database PGP (http://peppergenome.snu.ac.kr), and the InDel markers were located on chromosomes using MapDraw^41^. The genomic information of *C. annuum*, *C. chinense* and *C. baccatum* were also downloaded from PGP. The *C. annuum* genome was compared to *C. chinense* and *C. baccatum* genomes using the MCScan toolkit (V1.1)^42^. To determine synteny blocks, we used all-against-all LAST^43^ and fettered the LAST hits with a distance cutoff of 20 genes, also requiring at least 4 gene pairs per synteny block. Python version of MCScan was performed to construct chromosome-scale synteny blocks plots (https://github.com/tanghaibao/jcvi/wiki/ MCscan-(Python-version).

### Functional annotation of genetic InDels

The genes of related InDels were identified by comparison with the reference genome of CM334. The functions of these genes were predicted through sequence alignment with NR, SwissProt, GO, COG, KEGG database by BLAST. The Functional annotation of these genes were determined based on the information of the Gene Ontology Consortium (http://geneontology.org/).

### Experimental validation of DNA polymorphism

The PCR was performed in 20-μl of reaction mixture containing 2 μl genetic DNA sample (40 ng), 10 μl 2x Taq Mastermix II (Tiangen, Beijing, China), 0.5 μM of each primer and amount of ddH_2_O. The thermal cycles include 94 °C for 3 min, 30 cycles of 94 °C for 30 s, 55 °C for 30 s and 72 °C for 40 s, with an extension 72 °C for 7 min. The PCR products were analyzed by 10% polyacrylamide gel electrophoresis and visualized with silver staining.

### Phylogenetic analysis

PCR amplifications were separated on gels and scored as absent (0) or present (1). PowerMarker version 3.25 (Liu and Muse 2005, http://statgen.ncsu.edu/powermarker/) was used to calculate the number of alleles per locus, major allele frequency, gene diversity, polymorphism information content (PIC) values, and classical *F* st values. PowerMarker was performed to calculate Nei’s distance (Nei *et al*. 1973). Then, the unrooted phylogeny was constructed using the file of Nei’s distance based on neighbor-joining method with the tree viewed using MEGA 5.0 (Tamura *et al*. 2007, http://www.megasoftware.net/).

## Results

### Identification of InDel polymorphisms between *C. annuum* cv. G29 and *C. frutecens* cv. PBC688

A total of 319,522,376 and 309,682,186 clean reads were generated for PBC688 and G29, respectively. Using the Burrows-Wheeler Alignment (BWA), 2.54 × 10^8^ and 2.79 × 10^8^ of the PBC688 and G29, respectively, obtained reads were mapped to the reference genome CM334. The mapping read depth was 11x for PBC688 and 12x for G29. The overall genome coverage was 94.0% for PBC688 and 97.5% for G29, with an average of 95.8%. For PBC688 and G29, 76.2% and 87.9% pair-end (PE) reads, and 3.2% and 2.2% single-end (SE) reads were mapped to the reference chromosomes corresponding to 2.96 Gb of CM334 (Table 2).

**Table 2:** Summary of the original sequencing data of PBC688 and G29.

Sample	Clean-reads	PE (%)	SE (%)	Map ratio (%)	Q20 (%)	Depth	Cover ratio (%)
PBC688	319,522,376	76.2	3.2	79.4	94.9	11	94
G29	309,682,186	87.9	2.2	90.1	94.9	12	97.5
Average	314,602,281	82.1	2.7	84.8	94.9	11.5	95.8

Genome-wide insertion/deletion polymorphisms were examined via GATK software. In total, 1,664,770 InDels were identified between PBC688 and CM334. These InDels were distributed across all the twelve chromosomes, varying from 168,460 on chromosome 09 to 88, 291 on chromosome 08. At the same time, we identified 533,523 InDels between G29 and CM334 that ranged from 82,799 on chromosome 11 to 13,647 on chromosome 08. The InDels between PBC688 and G29 included different InDels than those described above, and the number of InDels ranged from 173,195 on chromosome 11 to 86,696 on chromosome 8 (Table 3).

**Table 3:** InDel polymorphisms identified on individual chromosomes of *Capsicum*.

	CD(MB)	PBC688 versus CM334		G29 versus CM334		PBC688 versus G29	
		InDel number	Frequency (InDels/Mb)	InDel number	Frequency (InDels/Mb)	InDel number	Frequency (InDels/Mb)
Chr1	272.7	152473	559.1	66466	243.7	159094	583.4
Chr2	171.1	112170	655.5	40498	236.7	110357	644.9
Chr3	257.9	163193	632.8	44010	170.6	158889	616.1
Chr4	222.6	129116	580.1	27962	125.6	125802	565.2
Chr5	233.5	135960	582.3	35179	150.7	134106	574.4
Chr6	236.9	141153	595.8	40996	173.0	137156	578.9
Chr7	231.9	145457	627.2	57444	247.7	140859	607.4
Chr8	145.1	88291	608.5	13647	94.1	86696	597.5
Chr9	252.8	146724	580.4	52697	208.5	150116	593.9
Chr10	233.6	143004	612.2	41440	177.4	138197	591.6
Chr11	259.7	168460	648.6	82799	318.8	173795	669.1
Chr12	235.7	138769	588.8	30385	128.9	136789	580.4
Total	2753.5	1,664,770	604.6	533,523	193.8	1,651,856	599.9

The average densities of the detected InDels between CM334 with PBC688 and G29 were 604.6 and 193.8 InDels/Mb, respectively. The InDels frequencies ranged from 655.5 InDels/Mb on chromosome 02 to 559.1 InDels/Mb on chromosome 01 between PBC688 and CM334, from 318.8 InDels/Mb on chromosome 11 to 94.1 InDels/Mb on chromosome 08 between G29 and CM334, and from 669.1 InDels/Mb on chromosome 11 to 563.2 InDels/Mb on chromosome 04 between PBC688 and G29 (Table 3).

In the present study, we detected that the largest InDel was 49 bp and the single base-pair InDels were dominant and accounted for about 65% of those analyzed. The ratios of InDels less than 10 bp were 94.4%, 92.6% and 94.3%, and those of less 6 bp was 89.1%, 86.2% and 89.1%, respectively, among the three different genomes (Table 4).

**Table 4:** The number and distribution ratios of InDels identified in the *Capsicum* genome.

InDel size (bp)	PBC688 versus CM334		G29 versus CM334		PBC688 versus G29	
	InDel number	Ratio (%)	InDel number	Ratio (%)	InDel number	Ratio (%)
1	1133853	68.1	345796	64.8	1129627	68.4
2	193287	11.6	62199	11.7	186832	11.3
3	79302	4.8	25317	4.7	79602	4.8
4	49406	3.0	16860	3.2	49560	3.0
5	26706	1.6	9614	1.8	27140	1.6
6	25864	1.6	9431	1.8	25056	1.5
7	16295	1.0	6322	1.2	15470	0.9
8	16777	1.0	6475	1.2	16107	1.0
9	16396	1.0	6348	1.2	15547	0.9
10	13945	0.8	5459	1.0	13361	0.8
≥11	92,939	5.6	39702	7.4	93554	5.7
Total	1664770	100.0	533523	100.0	1651856	100.0

### Genomic annotation and synteny of InDels in pepper

The use of the annotated genome of CM334 enabled the annotation of InDels, and to assign them with corresponding genes. We examined the distribution of the InDels related to genes of *Capsicum* and found that most of them were located within intergenic regions. Among the 1,664,770 and 533,523 InDel polymorphisms detected in CM334 compared with PBC688 and G29, 63,992 (3.8%) and 23,897 (4.5%) InDels were in gene regions, and only 2,519 and 1,019 were found in coding sequences. Among the 1,651,856 InDels identified between PBC688 and G29, 58,944 (3.6%) InDels were in genetic regions, with only 2,252 in coding sequences (Table 5).

**Table 5:** Location and types of InDel polymorphisms identified in *Capsicums*.

Region	Type	G108 vs CM334	G29 vs CM334	PBC688 vs G98
—	Intergenic	1571746	499518	1565544
—	Intragenic (without transcript)	57	1	57
—	Intron	4333	1547	4049
—	Upstream (within 5 Kb)	1540	561	1450
—	Downstream (within 5 Kb)	55535	20765	51124
—	Splice Site Acceptor	8	1	7
—	Splice Site Donor	4	3	5
CDS	Start Lost	7	2	7
CDS	Frame Shift	1685	663	1555
CDS	Codon Insertion	287	147	211
CDS	Codon Deletion	262	98	257
CDS	Codon Change Plus Codon Insertion	107	49	73
CDS	Codon Change Plus Codon Deletion	155	54	140
CDS	Stop Gained	10	5	6
CDS	Stop Lost	2	1	3
—	Other	29032	10108	27368
Total		1664770	533523	1651856

**Figure 1:**
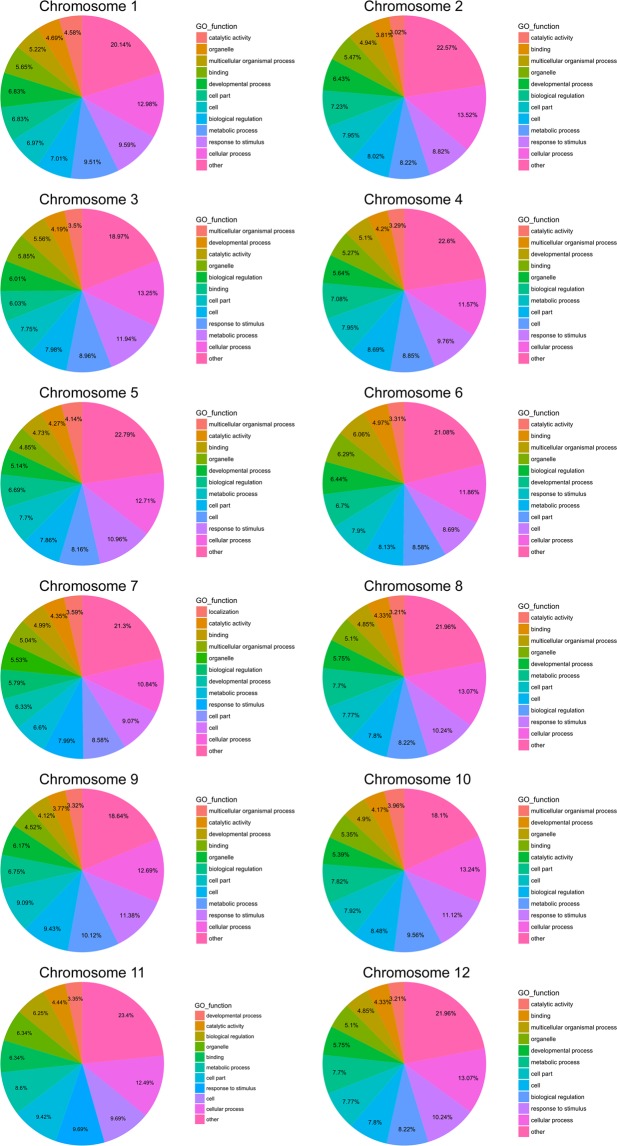
Chromosome annotation of polymorphic genic InDels associated with functional genes between PBC688 and G29.

The functional characterization of genes with the polymorphic InDels were distributed across all 12 chromosomes of pepper. Overall, most of the genes widely involved in cellular process, cell, cell part, metabolic process, response to stimulus, developmental process, biological regulation, organelle, multicellular organismal process, binding, catalytic activity, location and others (Fig. 1). Specifically, cellular process related genes consisted of most polymorphic InDels in all of chromosomes. Moreover, response to stimulus genes with high polymorphic InDels consisted of numerous polymorphic InDels in chromosome 1, 2, 4, 5, 8, 9 and 12. In chromosome 6, 7 and 11, the genes associated with cell (cellular component) consisted of more polymorphic InDels followed cellular process. However, in chromosome 3, genes referred to metabolic process involved in abundant InDels. In addition, most of genes have multiple functions and involve in regulation of multiple process (Supplementary Dataset 4).

**Figure 2:**
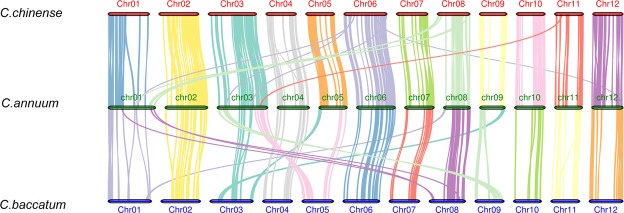
Syntenic blocks in the *C. annuum*, *C. chinense* and *C. baccatum* show the genome rearrangements among the three species.

Based on the three published genomes of *C. annuum, C. chinense and C. baccatum*, we analyzed the genetic synteny among them. In the *C. annuum* genome, we identified 202 and 131 syntenic blocks, involving 7,186 and 4,666 genes compared with *C. chinense* and *C. baccatum*, respectively (Supplementary Dataset 1 and 2). We found 106 and 60 chromosomal translocations between *C. annuum* to *C. chinense* and *C. baccatum*, respectively. However, these translocations were distributed on different chromosomes and could be used as firm evidence for chromosomal rearrangements. We found the translocations were located on different chromosomes between *C. annuum* and *C. chinense*: Chr01/Chr06, Chr01/Chr08, Chr03/Chr06, Chr03/Chr11, and Chr12/Chr06. Compared with *C. annuum* and *C. chinense*, translocations were located on more chromosomes between *C. annuum* and *C. baccatum*: Chr01/Chr08, Chr03/Chr05, Chr03/Chr09, Chr05/Chr03, Chr08/Chr01, Chr09/Chr03 (Fig. 2).

### Experimental validation of short InDel polymorphisms

**Figure 3:**
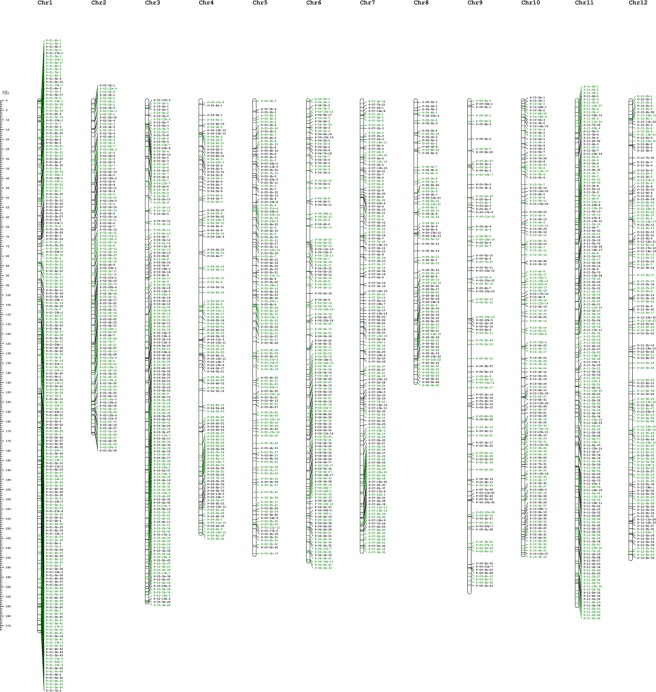
Distribution of 1605 InDels markers on each chromosome of the *C. capsicum* InDels marker names are listed to the right of the chromosomes. The ruler label to the left of chromosomes represents the physical distance. The black markers indicated deletion and red markers represented insertion.

To validate the InDels identified between PBC688 and G29, we selected 1605 out of 1,651,856 InDels following the rule of uniform distribution and converted them to PCR-based markers. According to the chromosomal location of InDels in *C. annuum* cv. CM334, the 1605 markers were distributed across all 12 chromosomes of pepper (Fig. 3 and Supplementary Dataset 3). Among the 1605 InDels, 69 (4.3%) InDels located to genetic regions (Supplementary Dataset 3). This rate was consistent with that of the whole genome. Then, we analyzed the genetic synteny of the blocks including 1605 InDels among the three published genomes of *Capsicum*. The *C. annuum* InDels shared highly conserved syntenic blocks with those of *C. chinense and C. baccatum* (Supplementary Fig. 1) improving the stability of these InDels among the different *Capsicum* species. Based on this selection, we designed primer pairs to amplify fragments of 150 bp surrounding the InDels. In the PCR analysis, most markers had clear amplification in PBC688 and G29 genomes with some others generating multiple amplicons.

For 1605 primer pairs of InDels, 1560 (97.2%) gave reliable amplification in PBC688 and G29. Using PAGE,1262 (78.6%) showed identifiable polymorphisms between PBC688 and G29; 90 of these produced an amplicon in only one genotype and therefore were not suitable for genetic analysis; 298 (18.6%) were monomorphic and 45 (2.8%) failed. The polymorphism rate increased slightly with increase of InDel length, and the polymorphism rate varied from 65.3% on InDels of 3 bp to 79.1% on those of more than 10 bp (Table 6).

**Table 6:** The distribution of polymorphic InDel markers between PBC688 and G29.

InDel size (bp)	InDels number	PBC688 versus G29			
		Codominant markers	Monomorphic markers	Dominant markers	No amplification
3	398	260 (65.3%)	104 (26.1%)	25 (6.3%)	9 (2.3%)
4	259	175 (67.6%)	66 (25.5%)	14 (5.4%)	4 (1.5%)
5	506	389 (76.9%)	72 (14.2%)	28 (5.5%)	17 (3.4%)
6–10	212	166 (78.3%)	26 (12.3%)	12 (5.7%)	8 (3.7%)
≥11	230	182 (79.1%)	30 (13.0%)	11 (4.8%)	7 (3.0%)
Total	1605	1172 (73.0%)	298 (18.6%)	90 (5.6%)	45 (2.8%)

To investigate the universal applicability of the InDel markers, we tested 288 among the inter-species and 576 between the intra-species. First, we screened five accessions representing five domesticated species for polymorphisms with 288 InDels. Polymorphisms were seen in 182 (63.2%) between PBC688 and G29 with 109 (37.8%) being monomorphic, while 194 (67.4%) and 87 (30.2%) were monomorphic among five accessions. Interestingly, twelve InDels monomorphic between PBC688 and G29 showed identifiable polymorphisms among five accessions. In addition, 7 (2.4%) produced no amplification in any accession. Together, our results suggest that these InDels may have universal applicability in the five domesticated species (Table 7). Then we selected two *C. annuum* accessions, G29 and G43, together with two *C. frutescens* accessions PBC688 and PI 439512 (16) to validate the InDel markers polymorphic between the intra-species accessions. Among 576 tested InDels (3–5 bp), 72 (12.5%) showed polymorphism between the two *C. annuum* accessions and 76 (13.2%) between the two *C. frutescens* accessions, although 488 (84.7%) were monomorphic between the two *C. annuum* accessions, 484 (84.0%) were monomorphic between the two *C. frutescens* accessions, and 16 (2.8%) failed in either species (Table 8).

**Table 7:** The distribution of polymorphic InDel markers among interspecific accessions.

InDel size (bp)	InDels number	PBC688 vs G29		2 vs 15 vs 24 vs 47 vs 60^a^		No amplification
		polymorphic InDels	Monomorphic InDels	polymorphic InDels	monomorphic InDels	
3	96	53 (55.2%)	40 (13.9%)	62 (64.6%)	31 (32.3%)	3 (3.1%)
4	96	61 (63.5%)	33 (11.5%)	66 (68.8%)	28 (29.2%)	2 (2.0%)
5	96	58 (60.4%)	36 (12.5%)	66 (68.8%)	28 (29.2%)	2 (2.0%)
total	288	182 (63.2%)	109 (37.8%)	194 (67.4%)	87 (30.2%)	7 (2.4%)

^a^2: *C. annuum* cv. PI 224408, 15: *C. frutescens* cv. PI 439512, 24: *C. chinense* cv. PI 441620, 47: *C. baccatum* cv. PI 441539, 60: *C. pubescens* cv. PI 585277.

**Table 8:** The distribution of polymorphic InDel markers between intraspecific accessions.

InDel size (bp)	InDel number	*C. annuum*		*C. frutescens*		No amplification
		G29 vs G43		PBC688 vs PI 439512		
		Polymorphism (Ratio)	Monomorphic (Ratio)	Polymorphism (Ratio)	Monomorphic (Ratio)	
3	192	22 (11.5%)	163 (84.9%)	26 (13.5%)	159 (82.8%)	7 (3.6%)
4	192	26 (13.5%)	161 (83.9%)	20 (10.4%)	167 (87.0%)	5 (2.6%)
5	192	24 (12.5%)	164 (85.4%)	30 (15.6%)	158 (82.3%)	4 (2.1.%)
Total	576	72 (12.5%)	488 (84.7%)	76 (13.2%)	484 (84.0%)	16 (2.8%)

### Experimental validation of the species-specific InDel markers

**Figure 4:**
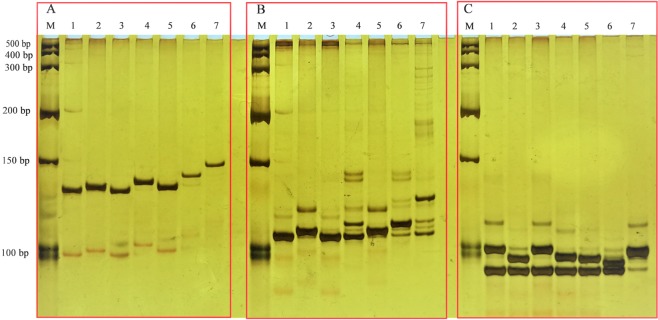
The PCR profiles of InDel-02-3b-22, InDel-02-3b-25 and InDel-03-3b-5 in 7 accessions representing 5 domesticated species (**A**) InDel-02-3b-25, (**B**) InDel-03-3b-5, (**C**) InDel-02-3b-22 M: Marker, 1: *C. annuum* cv. G29, 2: *C. frutescens* cv.PBC688, 3: *C. annuum* cv. PI 224408, 4: *C. frutescens* cv. PI 439512, 5: *C. chinense* cv. PI 441620, 6: *C. baccatum* var. *Pendulum* cv. PI 441539, 7: *C. pubescens* cv. PI 585277.

First, we found three InDel markers (InDel-02-3b-22, InDel-02-3b-25 and InDel-03-3b-5) each amplifying specific products in seven accessions representing five domesticated species (Fig. 4). To investigate the reliability of the result, we screened 10 accessions representing five domesticated species using these markers, and InDel-02-3b-22 and InDel-02-3b-25 revealed identifiable polymorphisms, while InDel-03-3b-5 amplified four specific products (Supplementary Fig. 2).

**Figure 5:**
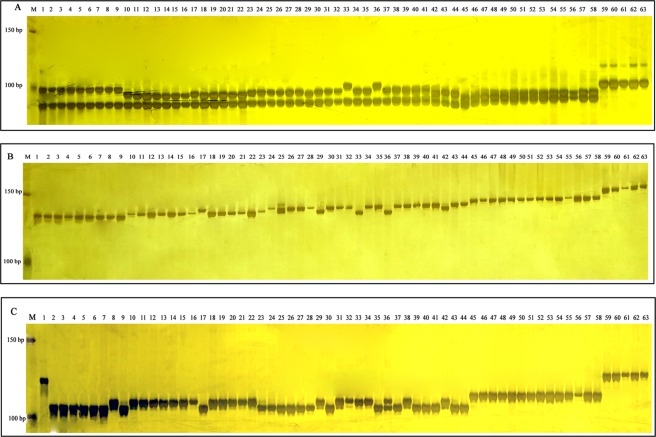
The PCR profiles of InDel-02-3b-22, InDel-02-3b-25 and InDel-03-3b-5 in 63 accessions representing 5 domesticated species (**A**) InDel-02-3b-22, (**B**) InDel-02-3b-25, (**C**): InDel-03-3b-5 M: Marker, 1-9: Nine accessions of *C. annuum*, 10–22: Thirteen accessions of *C. frutescens*, 23–44: Twenty-two accessions of *C. chinense*, 45–58: Fourteen accessions of *C. baccatum*, 59–63: Five accessions of *C. pubescens*.

**Figure 6:**
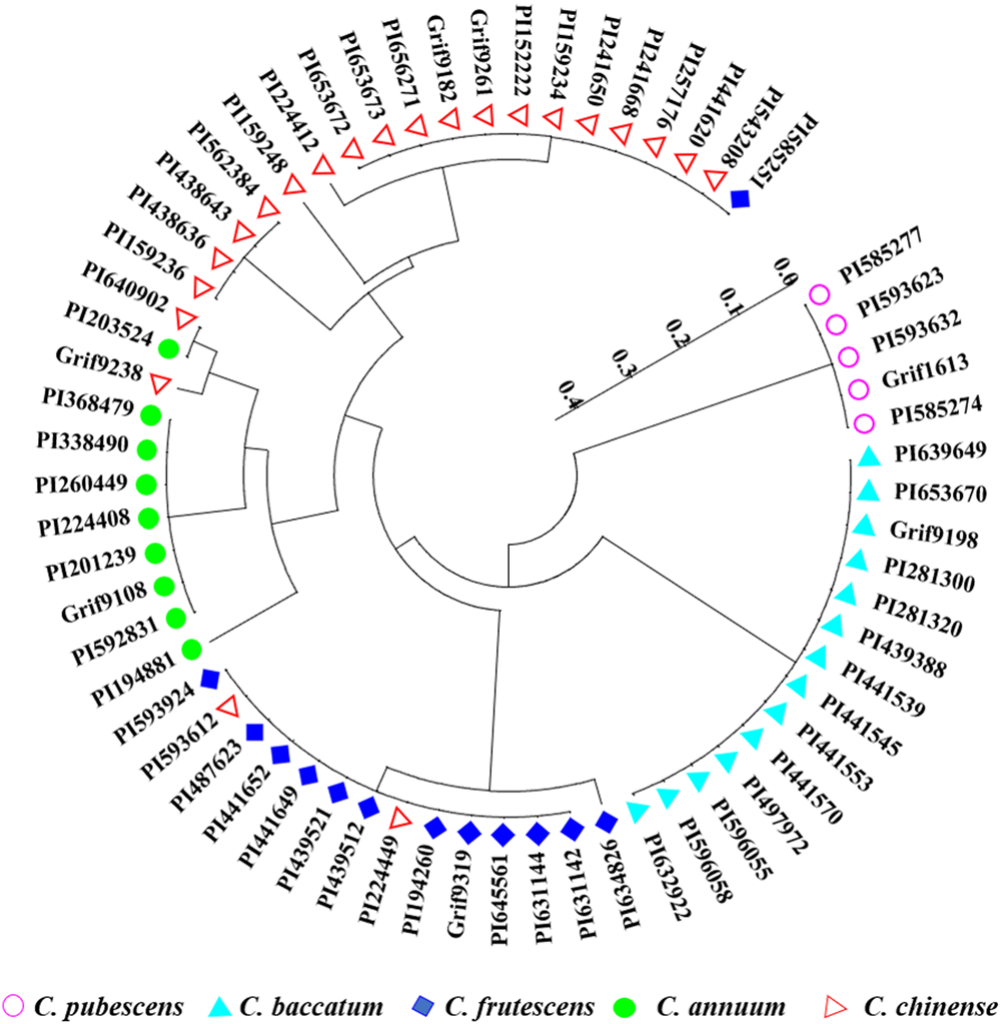
Phylogenetic tree based on the three InDel markers data showing the genetic relationship among the 63 *Capsicum* accessions.

To test whether InDel-02-3b-22 or InDel-02-3b-25 could individually distinguish five domesticated species, we randomly selected 63 accessions representing five domesticated species (Table 1). We detected 16 alleles for a total of 1008 data points through InDel analysis. The number of alleles at each locus varied from 5 for InDel-02-3b-22 and InDel-03-3b-5 to 6 for InDel-02-3b-25 (Fig. 5A–C, Supplementary Dataset 4). We used the variation for the 16 alleles to derive the dendrogram which showed that the 63 accessions were classified based on the five domesticated species. Among them, 58 accessions genotyped were consistent with the past subspecies classification. Specifically, nine *C.annuum*, fourteen *C. baccatum* and five *C. pubescens* were grouped into three classes. However, 2 of 22 *C. chinense* (PI593612 and PI224449) and 2 of 22 *C. chinense* (PI640902 and Grif9238) were grouped into the *C. frutescens* and *C.annuum* cluster, respectively. And 1 of 13 *C. frutescens* (PI585251) was grouped into the *C. chinense* cluster (Fig. 6). It is interesting that the three InDel markers InDel-02-3b-22, InDel-02-3b-25 and InDel-03-3b-5 associated with three genes, *CA02g13520*, *CA02g20590* and *CA03g07770*, respectively. Functional analysis showed *CA02g13520* encoded a protein with unknown function. *CA02g20590* encoded serine/threonine-protein kinase STY17-like. *CA03g07770* encoded the chloride channel protein CLC-d (Supplementary Dataset 3).

**Figure 7:**
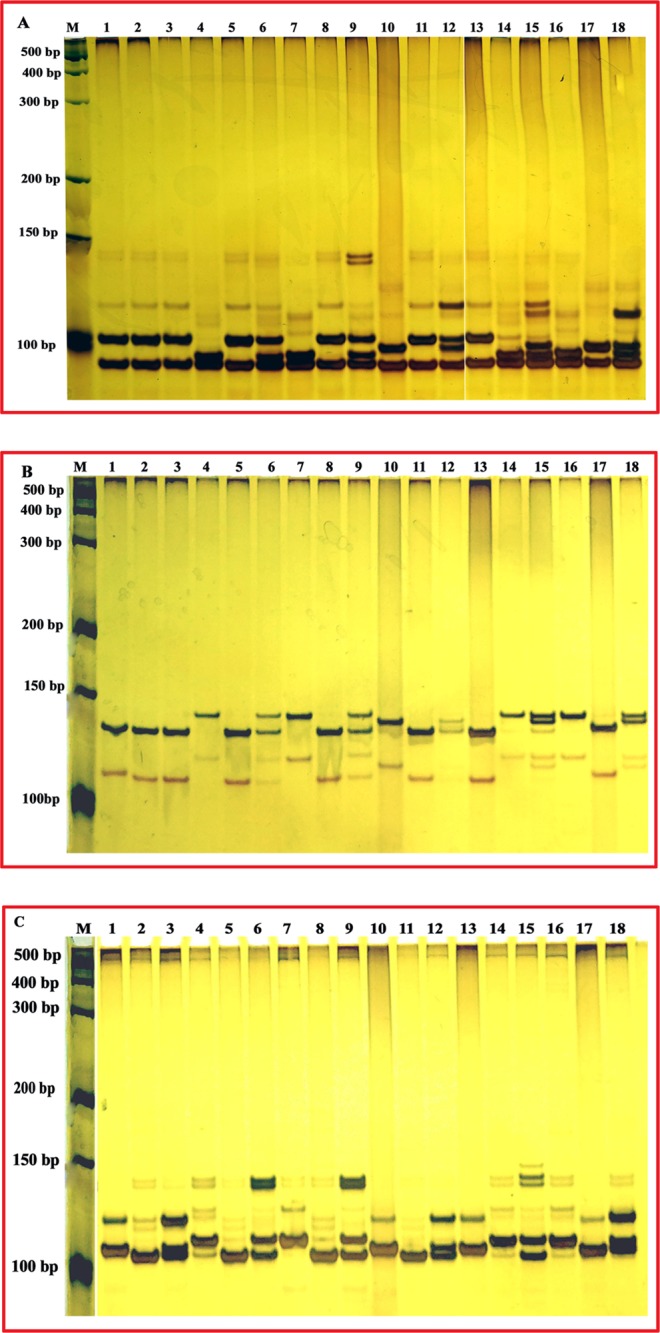
The PCR profiles of InDel-02-3b-22, InDel-02-3b-25 and InDel-03-3b-5 in 6 parents and their hybrids (**A**) InDel-02-3b-22, (**B**) InDel-02-3b-25, (**C**) InDel-03-3b-5 M: Marker 1–3: female parent: *C. chinense* cv. PI 640902, Male parent: *C. annuum* cv. G83, hybrid 4–6: female parent: *C. baccatum* cv. G568, Male parent: *C. annuum* cv. G83, hybrid 7–9: female parent: *C. baccatum* cv. PI441570, Male parent: *C. annuum* cv. G83, hybrid 10–12: female parent: *C.frutescens* cv. PI634826, Male parent: *C. annuum* cv. G83, hybrid 13–15: female parent: *C. chinense* cv. PI 159236, Male parent: female parent: *C. baccatum* cv. G568, hybrid 16–18: female parent: *C. baccatum* cv. PI441570, Male parent: female parent: *C. frutescens* cv. PI634826, hybrid.

To test the ability to identify the interspecific hybrids with three species-specific InDel markers, we selected six parents and their interspecific hybrids. We found that the fifth hybrid was incorrectly identified because its amplification pattern was not consistent with its parents with all three InDels (Fig. 7A–C). Either InDel-02-3b-22 or InDel-02-3b-25 could distinguish four of the remaining five hybrids, and InDel-03-3b-5 worked in all the cases (Fig. 7A–C). For the that hybrid that failed with InDel-02-3b-22 or InDel-02-3b-25, we found it was because these two markers could not differentiate its male parent *C. chinense* cv. PI 640902 and female parent *C. annuum* cv. G83. Our results imply that these three species-specific InDel markers could discriminate most hybrids formed from interspecific hybridization, and molecular markers are more accurate and convincing than phenotyping for identification.

## Discussion

Despite the development of SNP genotyping technologies, InDel markers also have important practical value for those researchers and breeders without the instruments to test SNP markers. We identified 1,651,856 InDels between PBC688 and G29 that represent an average of 599.9 InDels/Mb across the entire *Capsicum* genome. A previous study showed that the number of InDels from *C. annuum* cv. Perennial and cv. Dempsey was 654,158 and 694,494 respectively when compared with the CM334 genome sequence. However, the wild species *C. chinense* has a significantly higher level of InDels (2,450,533) compared to these two cultivars^34^. This is consistent with our study in that the number of InDels among *C. annuum* intra-species is quite low; in contrast, there exists a higher level of InDels among *Capsicum* inter-species. However, the number of InDels from the previous study was obviously less than that in our study. Approximately 555,400 short InDels (1–5 bp) were detected in Zunla-1 relative to Chiltepin, and, 373,785 and 231,056 short InDels (1–5 bp) were detected in Zunla-1 relative to *C. chinense* and CM334^2^. There may be two main reasons for the difference. Firstly, in our study, we used CM334 genome as the reference genome, so our results are consistent with the study. Secondly, the previous study only detected short InDels (1–5 bp), so the number of InDels was significantly less than that in our study.

Chromosomal rearrangement often produces unbalanced gametes that reduce hybrid fertility and plays an important role in promoting speciation^44^. In our study, collinearity comparison among *Capsicum* species revealed that chromosomes 1, 3, 5, 8, 9 and 12 exhibit translocations that differentiate *C.annuum* from *C.chinense* and *C.baccatum*. Our result was similar with previous studies about *Capsicum* species. Kim *et al*. reported that chromosomal translocations among chromosomes 3, 5, and 9 were observed by comparison between *C.baccatum* and the two other peppers^45^. Wu *et al*. reported the cultivated *C.annuum* genome included two acrocentric chromosomes versus a single acrocentric chromosome detected in *C. chinense*, *C. frutescens* and wild *C.annuum*^46^. Moreover, Wu *et al*. revealed that between the pepper and tomato genomes there exists at least 19 inversions, 6 chromosome translocations, and numerous putative single gene transpositions as determined by collinearity comparison^46^. Based on the genomes of *Capsicum* species and two *Solanum* species, collinearity comparisons showed that chromosome 6 and 4 of *Solanum* were discovered in the terminal regions of the long and short arms of chromosomes 3 and 5 in *C.annuum* and *C.chinense*, respectively^45^.

In this study, the localization of InDels within the pepper genome showed more than 95% InDels were in intergenic regions. Similarly, more InDels were detected in the intron than in CDS. Previous studies about genome-wide SNP and InDel discovery revealed the similar results in multiple crops, such as tomato and *Brassica rapa*^31,47^. In pepper, 93.06% and 93.39% of intergenic SNPs were detected for varieties PRH1 and Saengryeg, respectively^48^.

In order to obtain in-depth knowledge in the InDels in our study associated with genes, these polymorphic InDels within genetic regions were functionally annotated in each chromosome. The current results revealed that genes involved in cellular process consisted of most polymorphic InDels in all chromosomes. Then, high polymorphic InDels with “response to stimulus” related genes InDelwere mapped in chromosomes 1, 2, 4, 5, 8, 9 and 12. Because of different focus, our results had some differences with a previous study by Ahn *et al*., who reported that most genes with high polymorphic SNPs were related with carbohydrate metabolism, followed by transcription regulation, ion binding and others. In addition, they found numerous genes with high polymorphic SNPs related to disease resistance mapped to chromosome 4, which could play a vital role in future pepper breeding^47^.

In this study, we confirmed InDels can be developed as potentially valuable genetic markers with a reliable high rate of polymorphism. Among 1605 InDels of 3–49 bp in length, 1262 (78.6%) showed polymorphisms. Only 45 (2.8%) of the primers yielded no amplification from either of the two sequenced accessions. This can be explained by sequence variations in the primer binding sites among *Capsicum* species as we designed primers based on the reference genome sequence^31^. In contrast to the high polymorphism rate of InDels among five accessions representing five domesticated species, two *C. annuum* and *C. frutescens* accessions showed much lower polymorphism rates. As expected, our results suggest that polymorphism rate of InDel markers within species was much lower than that among species. In a previous study on genome-wide re-sequencing inbred lines *C. annuum* cv. BA3 and B702, more than 90% of the InDel markers were amplified. However, only 27.2% and 12.9% markers were polymorphic between BA3 with B702 or *C. frutescens* cv. YNXML, respectively^9,27^.

Most importantly, we found three inter-species specific InDels (InDel-02-3b-22, InDel-02-3b-25 and InDel-3b-3-5) each of which could highly discriminate among most of the accessions under study and which efficiently identified interspecific hybrids, implying their potential application for new germplasm classification and interspecific hybrid identification in the future. Our results showed that InDel-02-3b-22, InDel-02-3b-25 and InDel-03-3b-5 could individually discriminate almost all the accessions, which agrees with a previous study. Di Dato *et al*. (2015) showed that most accessions (among 59 accessions) were clearly differentiated with ten SSR markers except two accessions of *C. chinense*, which were grouped into *C. frutescens* cluster. He concluded that the two abnormal accessions were genetically distant from others analyzed *C. chinense*^12^. In our study, the accessions of *C.annuum*, *C. baccatum* and *C. pubescens* had clearly specific amplification products, although 4 accessions of *C. chinense* and 1 accessions of *C. frutescens* showed some confusing patterns. Our results confirmed previous findings based on both phenotypes and molecular markers that *C. annuum* was closely related to *C. chinense* and *C. frutescens*, and distant to *C. baccatum* and *C. pubescens*^12,49^.

The location of markers is a vital factor for the application value of markers. These markers are located in intragenic regions to implicate the phenotypic traits and have more potential applications in marker assisted selection as functional markers^4^. In our study, the three InDel markers InDel-02-3b-22, InDel-02-3b-25 and InDel-03-3b-5 were in intragenic regions and associated with three genes, *CA02g13520*, *CA02g20590* and *CA03g07770*, respectively. *CA02g20590* encoded serine/threonine-protein kinase STY17-like. In *Arabidopsis thaliana*, the protein kinases STY8, STY17, and STY46 played a vital role in phosphorylating of transit peptides for chloroplast-destined preproteins^50^. *CA03g07770* encoded the chloride channel protein CLC-d. In *Arabidopsis thaliana*, CLCd was targeted to Golgi apparatus and could suppress the cation-sensitive phenotype of Δ gef1^51^. Although *CA02g13520* encodes a protein with unknown function, but it can be applied to marker assisted selection as a functional marker without any effect.

Together, these novel InDel markers are very valuable reference tools for classification of germplasm resource, identification of interspecific hybrids, genetic research, and marker-assisted breeding in pepper.

## Supplementary information

Supplementary information

Supplementary datasets
