# Supplementary material for: Development and Application of InDel Markers for Capsicum spp. Based on Whole-Genome Re-Sequencing
Source: Sci Rep. 2019 Mar 6;9:3691. doi: 10.1038/s41598-019-40244-y (PMC6403297; doi:10.1038/s41598-019-40244-y)

Title: Development and Application of InDel Markers for *Capsicum Spp* Based on Whole-Genome Re-Sequencing

Running title: Development and Application of InDel Markers in pepper

Authors byline:

Guangjun Guo[1], Genlian Zhang[1,2], Baogui Pan[1], Weiping Diao[1], Jinbing Liu[1], Wei Ge[1], Changzhou Gao[1], Yong Zhang[3], Cheng Jiang[3], Shubin Wang[1*]

Authors' addresses:

1. *Institute of Vegetable Crops, Jiangsu Academy of Agricultural Sciences / Jiangsu Key Laboratory for Horticultural Crop Genetic Improvement, Nanjing, Jiangsu, 210014 China*
2. *College of Horticulture, Nanjing Agricultural University, Nanjing 210095, China*
3. *College of Horticulture, Henan Agricultural University, Zhengzhou 450002, China*

Authors' Email:

Guangjun Guo: ggj-198@163.com

Genlian Zhang: 15261872273@163.com

Baogui Pan: pantix@163.com

Weiping Diao: diaowp_2000@163.com

Jinbing Liu: pepprljb@163.com

Wei Ge: gewei_127@163.com

Changzhou Gao: gaochangzhou8@163.com

Yong Zhang: 1404703118@qq.com

Cheng Jiang: 1144982957@qq.com

Corresponding author:

Dr. S. B. Wang**;** Institute of Vegetable Crops, Jiangsu Academy of Agricultural Sciences/Jiangsu Key Laboratory for Horticultural Crop Genetic Improvement, Nanjing, Jiangsu, 210014 China; telephone, +86-025-84390265; Fax, +86-025-84390262; E-mail, wangsbpep@163.com

Supplementary dataset 1 Identification of the synteny blocks between C. annuum and C.chinense

Supplementary dataset 2 Identification of the synteny blocks between C. annuum and C.baccatum

Supplementary dataset 3 The information of 1605 InDels markers

Supplementary dataset 4 Chromosome annotation of polymorphic genic InDels associated with functional genes between PBC688 and G29

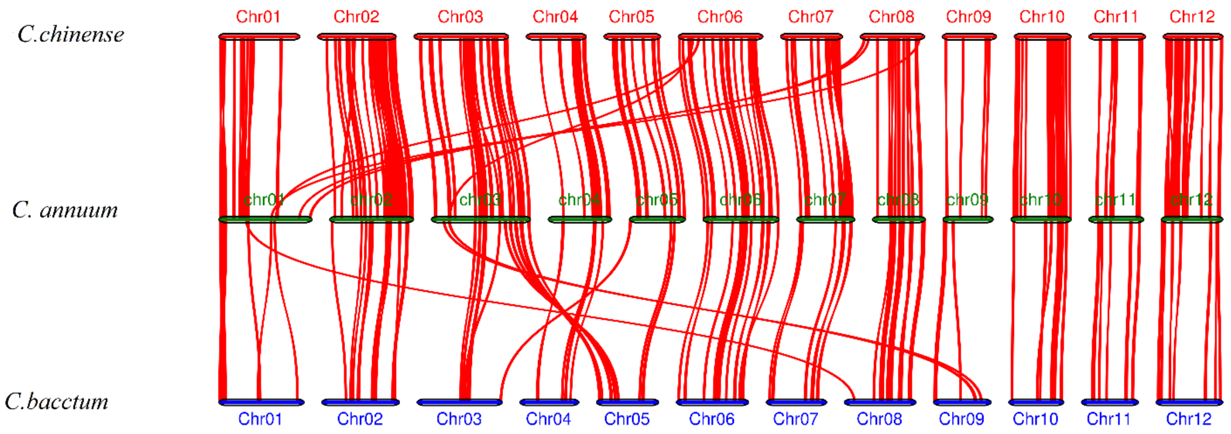

Supplementary Figure 1 Syntenic blocks including the 1605 InDels in the *C.annuum*, *C.chinense* and *C.baccatum* show that genome rearrangements have occurred among these species.

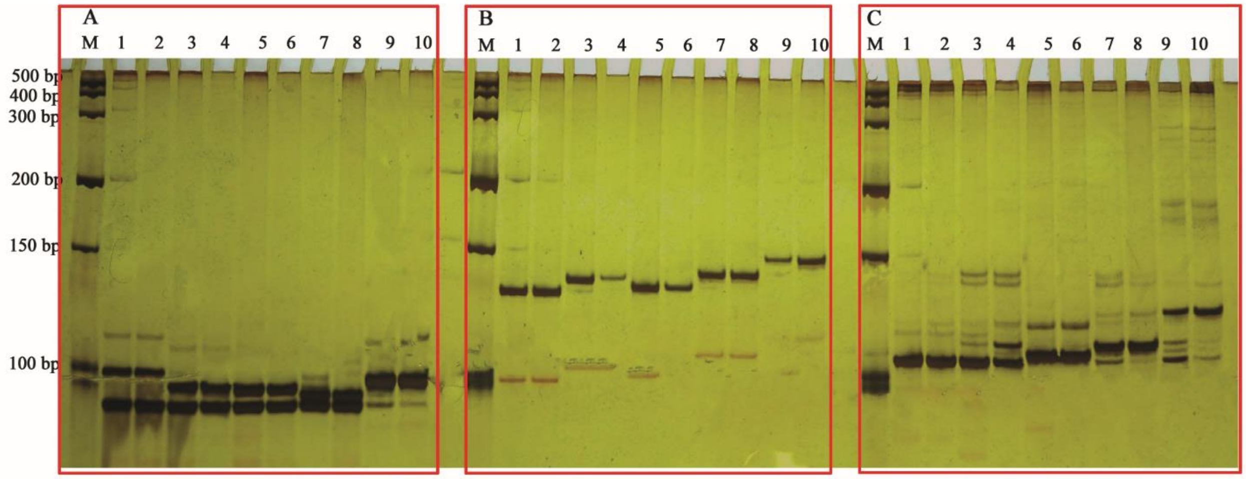

Supplementary Figure 2 The PCR profiles of InDel-2-22, InDel-2-25 and InDel-3-5 in 10 accessions representing 5 domesticated species

A: InDel-2-22, B: InDel-2-25, C: InDel-3-5

M: Marker, 1 and 2: *C.annuum* cv. PI 368479 and PI 260449, 3 and 4: *C.frutescens* cv. PI 441649 and PI 631144, 5 and 6: *C.chinese* cv. PI 152222 and PI 257176, 7 and 8: *C.baccatum* var. *Pendulum* cv. PI 441539 and *C.baccatum* PI 439388, 9 and 10: *C.pubescens* cv. PI 585277 and Grif 1613

Full-length gels

Figure 4

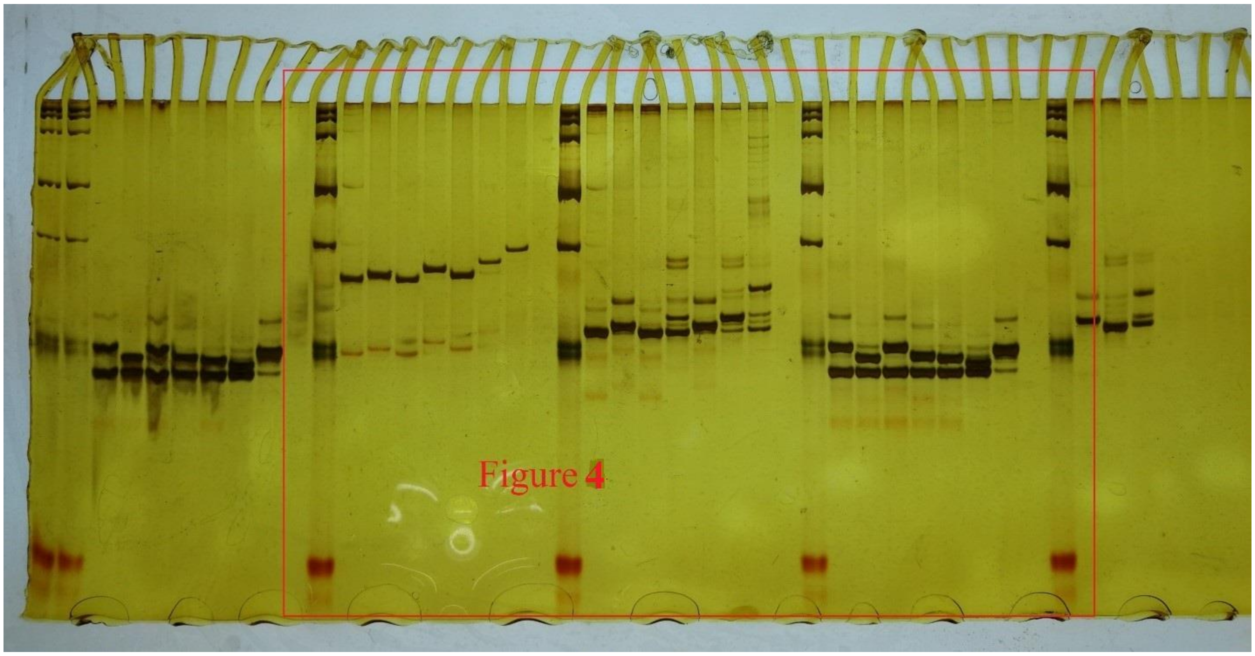

Figure 5A

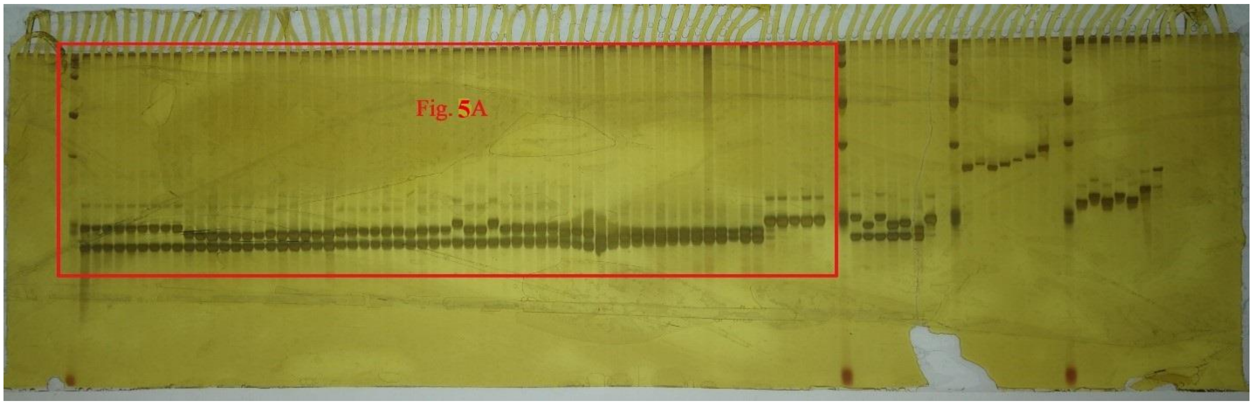

Figure 5B

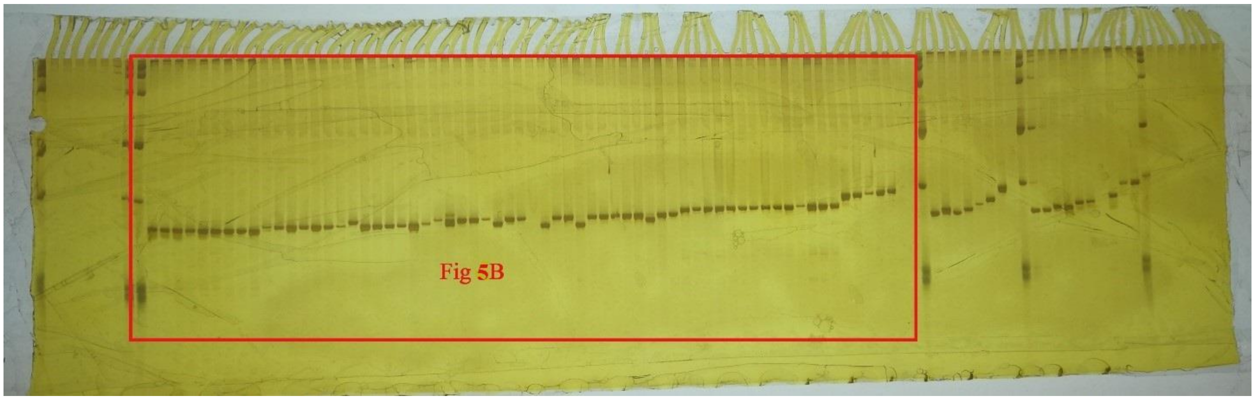

Figure 5C

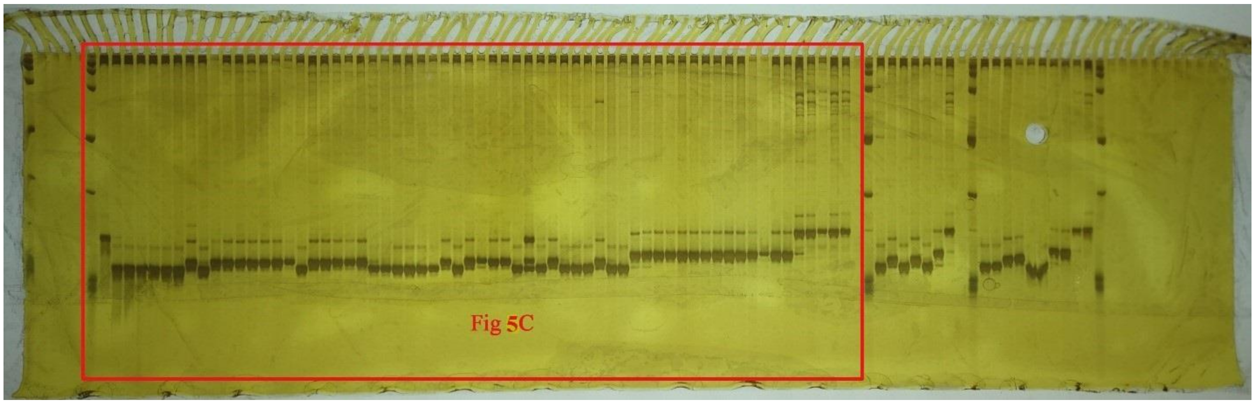

Figure 7

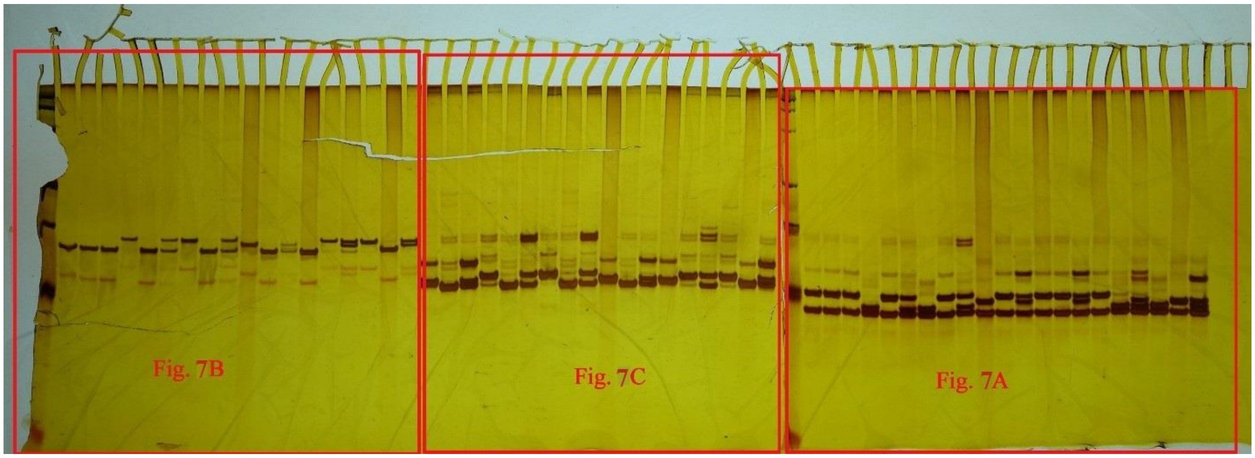

Supplement: Supplementary file 1 — Supplementary information [file 41598_2019_40244_MOESM1_ESM.pdf]
